# Chemical Inhibition of Apurinic-Apyrimidinic Endonuclease 1 Redox and DNA Repair Functions Affects the Inflammatory Response via Different but Overlapping Mechanisms

**DOI:** 10.3389/fcell.2021.731588

**Published:** 2021-09-20

**Authors:** Thais Teixeira Oliveira, Fabrícia Lima Fontes-Dantas, Rayssa Karla de Medeiros Oliveira, Daniele Maria Lopes Pinheiro, Leonam Gomes Coutinho, Vandeclecio Lira da Silva, Sandro José de Souza, Lucymara Fassarella Agnez-Lima

**Affiliations:** ^1^Departamento de Biologia Celular e Genética, Universidade Federal do Rio Grande do Norte, UFRN, Natal, Brazil; ^2^Instituto Federal de Educação Tecnológica do Rio Grande do Norte, IFRN, São Paulo do Potengi, Brazil; ^3^Instituto do Cérebro, Universidade Federal do Rio Grande do Norte, Natal, Brazil; ^4^Bioinformatics Multidisciplinary Environment (BioME), IMD, Universidade Federal do Rio Grande do Norte, Natal, Brazil

**Keywords:** apurinic/apyrimidinic endonuclease I (APE1), DNA repair, transcriptional control, inflammation, ETS transcription factor

## Abstract

The presence of oxidized DNA lesions, such as 7,8-dihydro-8-oxoguanine (8-oxoG) and apurinic/apyrimidinic sites (AP sites), has been described as epigenetic signals that are involved in gene expression control. In mammals, Apurinic-apyrimidinic endonuclease 1/Redox factor-1 (APE1/Ref-1) is the main AP endonuclease of the base excision repair (BER) pathway and is involved in active demethylation processes. In addition, APE1/Ref-1, through its redox function, regulates several transcriptional factors. However, the transcriptional control targets of each APE1 function are not completely known. In this study, a transcriptomic approach was used to investigate the effects of chemical inhibition of APE1/Ref-1 redox or DNA repair functions by E3330 or methoxyamine (MX) in an inflammatory cellular model. Under lipopolysaccharide (LPS) stimulation, both E3330 and MX reduced the expression of some cytokines and chemokines. Interestingly, E3330 treatment reduced cell viability after 48 h of the treatment. Genes related to inflammatory response and mitochondrial processes were downregulated in both treatments. In the E3330 treatment, RNA processing and ribosome biogenesis genes were downregulated, while they were upregulated in the MX treatment. Furthermore, in the E3330 treatment, the cellular stress response was the main upregulated process, while the cellular macromolecule metabolic process was observed in MX-upregulated genes. Nuclear respiratory factor 1 (NRF1) was predicted to be a master regulator of the downregulated genes in both treatments, while the ETS transcription factor ELK1 (ELK1) was predicted to be a master regulator only for E3330 treatment. Decreased expression of ELK1 and its target genes and a reduced 28S/18S ratio were observed, suggesting impaired rRNA processing. In addition, both redox and repair functions can affect the expression of NRF1 and GABPA target genes. The master regulators predicted for upregulated genes were YY1 and FLI1 for the E3330 and MX treatments, respectively. In summary, the chemical inhibition of APE1/Ref-1 affects gene expression regulated mainly by transcriptional factors of the ETS family, showing partial overlap of APE1 redox and DNA repair functions, suggesting that these activities are not entirely independent. This work provides a new perspective on the interaction between APE1 redox and DNA repair activity in inflammatory response modulation and transcription.

## Introduction

Apurinic-apyrimidinic endonuclease 1/Redox factor-1 (APE1/Ref-1) is a multifunctional protein involved in cell growth, transcriptional regulation, stress response, and genome stability. Two functionally distinct domains exert the biological activities of APE1/Ref-1. The N-terminal domain contains a nuclear localization signal. It is associated with the redox activity of APE1/Ref-1, while the C-terminal contains an endonuclease domain involved in the repair of abasic sites (or AP sites) in DNA (Xanthoudakis et al., 1994; Tell et al., 2010; Li and Wilson, 2014; Antoniali et al., 2017). The redox function activates transcription factors, such as AP-1 and NF-κB, which influence cellular processes such as stress responses, DNA repair, angiogenesis, and cell survival (Ando et al., 2008; Lee et al., 2009; Jedinak et al., 2011; Kelley et al., 2012). It also exhibits a redox-independent transcriptional regulatory function, acting as a transcriptional repressor by binding to negative Ca^2+^ response elements (nCaRE) (i.e., parathyroid hormone and *APEX1* promoters), which allows APE1/REF-1 to regulate its expression (Izumi et al., 1996; Kuninger, 2002). APE1/Ref-1 is also associated with transcription factors and other co-activators, which are required to assemble pre-initiation complexes and regulate transcription in a redox-independent manner (Fantini et al., 2008; Sengupta et al., 2012).

In addition to the role of APE1/Ref-1 in transcriptional regulation, the transcriptional role of base excision repair (BER) enzymes, including APE1/Ref-1, has recently emerged in both active demethylation processes mediated by ten-eleven translocation (TET) and thymine DNA glycosylase enzymes (Jin et al., 2015; Bochtler et al., 2016). The repair of 8-oxoguanine (8-oxoG) in promoter regions has also been described as an epigenetic mechanism (Ba et al., 2014; Fleming and Burrows, 2017). It has been demonstrated that the recruitment of APE1/Ref-1 and OGG1 (8-Oxoguanine DNA Glycosylase) to oxidized lesions generated by lysine-specific demethylase 1 (LSD1) activity on promoters, is required for further binding of transcription factors (TFs), such as c-Myc, and stabilization of the transcriptional complexes (Amente et al., 2010). In addition, the presence of DNA modifications, such as 8-oxoG and AP sites, in gene promoters has been related to increased gene expression (Pan et al., 2016; Fleming and Burrows, 2017). Recent studies have shown that the redox and repair activities of APE1/Ref-1 can affect the expression of the same genes (Li et al., 2019). Despite these vital functions, the targets and phenotypes associated with transcriptional control exercised by redox or DNA repair functions of APE1/Ref-1 are poorly understood.

Previously, we demonstrated the association between polymorphisms in *OGG1*, *PARP-1*, and *APEX1* with bacterial meningitis. In addition, the patient’s carriers of *APEX1* 148 Glu allele presented reduced expression of cytokines and chemokines, such as IL-6, MCP-1, and IL-8, and an increase in DNA damage level, suggesting that APE1/Ref-1 repair activity is affected. Thus, these data indicate that DNA repair activity may be involved in these mechanisms (da Silva et al., 2011).

Deregulated APE1/Ref-1 is associated with various human pathologies, such as cancer, atherosclerosis, neurodegeneration, and infectious diseases, making it a potential therapeutic target (Thakur et al., 2014; Shah et al., 2017). Several research groups have collaborated to identify molecular inhibitors of APE1/Ref-1 activity. Quinone (E)-3-(2-[5,6-dimethoxy-3-methyl-1,4-benzoquinonyl])-2-nonyl propanoic acid (E3330) has therapeutic potential as a specific and direct redox inhibitor of APE1/Ref-1, as it acts like H_2_O_2_ by increasing Cys-65/93/99 oxidation (Kelley et al., 2011; Luo et al., 2012; Cesaratto et al., 2013; Zhang et al., 2013). Some studies have shown that E3330 decreases the expression of inflammatory modulators, such as tumor necrosis factor-α (TNF-α) and interleukin IL-8, and inhibits the growth and migration of cancer cells (Fishel et al., 2011; Kelley et al., 2011; Su et al., 2011; Li and Wilson, 2014; Ding et al., 2017).

The inhibition of APE1/Ref-1 DNA repair activity is associated with the sensitization of cancer cells to chemotherapy (Bobola et al., 2005; McNeill et al., 2009). Methoxyamine (MX), a synthetic molecule designed to inhibit BER, binds to high affinity to the aldehyde groups of AP sites, which are chemically refractory to APE1/Ref-1 endonuclease activity and resistant to BER processing (Rosa et al., 1991; Wilson and Simeonov, 2010). The inhibition of AP endonuclease activity of APE1/Ref-1 by MX has been studied in association with chemotherapeutic drugs, and positive results have been obtained (Liu et al., 2003; Guerreiro et al., 2013; Montaldi et al., 2015; Laev et al., 2017).

Although the APE1/Ref-1 redox function in transcriptional regulation of inflammatory mediators is known, the impact of E3330 and MX on the transcriptional regulation during inflammatory response is mediated by the inhibition of APE1/Ref-1 activities is unknown, and it is exploited in this survey. The present study analyzed the cell transcriptome profile of a lymphoma-derived monocyte cell line (U937) stimulated with lipopolysaccharide (LPS) to investigate APE1/Ref-1 redox and repair activities on transcriptional regulation during the inflammatory response. We found that the expression of inflammatory modulators was reduced in this model after treatment with both APE1/Ref-1 activity inhibitors. Furthermore, comparative transcriptome analysis revealed that genes related to inflammatory responses and mitochondrial processes were downregulated in both treatments. However, the treatments differed in terms of rRNA processing and ribosome biogenesis. We also predicted master regulators to differentially expressed genes and identified NRF1, YY1, and the ETS family of TFs as the most likely APE1/Ref-1 partners in inflammatory signaling in monocytes.

## Materials and Methods

### Cellular Model of Inflammation

U937 monocyte-like cells, derived from patients with histiocytic lymphoma (ATCC^®^ CRL1593.2), were cultured in Gibco RPMI-1640 medium (Thermo Fisher Scientific Inc., Waltham, MA, United States) supplemented with 44 mM sodium bicarbonate (Sigma-Aldrich, St. Louis, MO, United States), Gibco 10% fetal bovine serum (Thermo Fisher Scientific Inc.), and 1% Penicillin-Streptomycin antibiotic solution (Sigma-Aldrich) in a humidified incubator at 37°C and a 5% CO_2_ atmosphere unless stated otherwise. Cells (5 × 10^5^) in 3 mL of medium were stimulated with 1 μg/mL LPS (Cat. No: L2654; Sigma-Aldrich) for 24 h, and then incubated with 100 μM E3330 (≥98% pure; Sigma-Aldrich) or 6 mM methoxyamine (MX) for 4 h. The cells were grouped as follows: unstimulated (control), LPS-stimulated (LPS), LPS + 100 μM E3330 (LPS + E3330), and LPS + 6 mM MX (LPS + MX). We ensured that the cell lines used in these experiments were free of mycoplasma infection.

### Cell Viability Assays

For U937 cell viability measurement, 5 × 10^5^ cells from each experimental group were incubated for 2, 4, 6, 24, and 48 h and subjected to trypan blue exclusion assay. In addition, the LPS + MX and LPS + E3330 groups were pre-treated with LPS for 1 h and co-incubated with MX and E3330. The cell suspension was mixed with Trypan blue dye 1:1 (v:v), and the cellular capacity to exclude the dye was analyzed using a hemocytometer and a CKX41 inverted microscope (Olympus Optical Co. Ltd., Tokyo, Japan). Cell viability was calculated as the difference between the dead and total cell counts. The data were obtained in triplicate.

### Cytokine and Chemokine Measurements

The proinflammatory cytokines and chemokines levels in U937 cells were measured using the Bio-Plex 200 suspension array system (Bio-Rad). The Human Cytokine/Chemokine Panel (MPXHCYTO-60k; EMD Millipore, Burlington, MA, United States) included the inflammatory modulators, TNF-α, IL-6, IL-10, MIP1α/CCL3, MIP-1β/CCL4, IL-8/CXCL8, and MCP1. Samples were processed and measured according to the manufacturer’s instructions. Cytokine/chemokine expression was measured in duplicate. Results were determined based on a parametric logistic equation using Bio-Plex Manager 4.01 software (Bio-Rad) and are expressed as picograms per milliliter.

### Apurinic/Apyrimidinic-Site Incision Assays

To determine if the 100 μM E3330 does not change the repair activity of APE1/Ref-1, we performed AP-site incision assays. An oligonucleotide gel-based APE1/Ref-1 endonuclease activity assay was performed as previously described by Silva-Portela et al. (2016) with modifications stated further in this section. The AP endonuclease activity of commercially available APE1 (NEB) was verified after treatment with E3330, based on the cleavage of a double-stranded DNA (dsDNA) substrate containing an abasic site at position 10 of the oligonucleotide 21-mer (5′-Cy5-CGGAATTAAAGXGCAAGACCT-3′ and 5′-AGGTCTTGCCCTTTAATTCCG-3′). This oligonucleotide was 5′-fluorescently labeled with Cy5. Standard reactions containing dsDNA (100 fmol), NEBuffer 4 (10×), and E3330, with or without APE1 (NEB), were incubated for 60 min at 37°C. The reactions were terminated by adding a “STOP” solution (98% formamide and 0.5 M EDTA) and heated to 95°C for 3 min. Samples (20 μL) were then run on a 20% polyacrylamide gel containing 8 M urea in 1×-TBE buffer at 300 V for 240 min. The reaction products were observed using a Chemidoc System (Bio-Rad).

### Chromatin Immunoprecipitation

Chromatin immunoprecipitation (ChIP) assays were performed using 10^6^ cells stimulated according to our study model. DNA was cross-linked with 1% paraformaldehyde and sheared (average 200 bp) with five cycles of 10-s fragmentation using an ultrasonic bath (Ultra 800, Ciencor Scientific Ltd.). Further, DNA protein complexes were immunoprecipitated with ChIP quality Abs (APE1/Ref-1, sc-17774, Santa Cruz Biotechnology) using the Chromatin Immunoprecipitation Assay kit (Merck Millipore). The precipitates were washed three times, de-cross-linked, and subjected to PCR. TNF4 promoter primers were used: (−335 to −228 bp) F 5′AGGCAATAGGTTTTGAGGGCCAT3′ and R 5′TCCTCCCTGCTCCGATTCCG3′.

### Immunofluorescence

We determined the subcellular localization of APE1 as follows. First, the cells were washed with phosphate-buffered saline (PBS), resuspended in 5 mL 3.7% paraformaldehyde in PBS for 15 min, washed with cold PBS, and seeded on coverslips that had been treated with poly-L-lysine for 30 min at room temperature. The cells were then incubated with Triton X-100 (0.5%) in PBS for 15 min and washed three times for 5 min each with Tween-20 (0.1%) in PBS (PBST). The cells were then incubated with anti-APE1 (sc-17774, Santa Cruz Biotechnology) for 1 h, washed three times with PBST, and incubated with FITC-conjugated secondary antibody for 1 h. Finally, the sections were washed and mounted with Dako solution + DAPI (1.5 μg/mL), and stained cells were visualized using a CKX41 fluorescence microscope (Olympus) attached to a DP70 fluorescence camera (Olympus). All the mentioned methods were performed in the dark at room temperature.

### RNA Extraction and cDNA Synthesis

Total RNA was isolated from U937 cells using IllustraTM RNAspin Mini RNA Isolation Kits (GE Healthcare Little Chalfont, United Kingdom), as described by the manufacturer. Messenger RNA was obtained using an mRNA isolation kit (Roche Holdings AG, Basel, Switzerland). Briefly, the mRNA poly(A)+ tails were hybridized to a biotin-labeled oligo(dT) probe, and streptavidin-coated magnetic particles captured the biotinylated dT-A hybrids. A magnetic particle separator collected the magnetic particles, which were washed to remove contaminants, and then mRNA was eluted from the particles with water. The quantity of recovered mRNA was assessed using a Bioanalyzer 2100 (RNA 6000 Nano; Agilent Technologies GmbH., Waldbronn, Germany) and quantified using Nanovue (GE Healthcare). Total RNA for quantitative PCR was extracted using IllustraTM triplePrep Kit (GE Healthcare), as described by the manufacturer. Complementary DNA was prepared from the extracted total RNA using High-Capacity cDNA Reverse Transcription Kits (Applied Biosystems), as described by the manufacturer.

### Transcriptome Analysis

Approximately 5 μg of complementary DNA from each group was sequenced using a 454 GS FLX Titanium, following the manufacturer’s protocol (Roche Holdings AG). The sequenced data were aligned against the Ref-Seq database of human expressed sequences provided by the University of California Santa Cruz (UCSC^1^), accessed on January 30, 2014. Sequences were aligned using the BLAT (Kent, 2002). The results were filtered using the pslCDnaFilter tool,^2^ with a minimum identity of 98%, minimum coverage of 90%, and only one alignment per sequence. All sequences that matched the ribosomal RNA (rRNA) were excluded. Gene expression was normalized as counts per million (CPM), calculated by counting reads per gene (Xi) and the total number of reads per sample (N): CPM = (Xi/N) × 10^7^. Thus, the fold change in each gene was estimated between the samples. The Ensembl Gene ID from downregulated (≤−2-fold change) and upregulated (≥2-fold change) transcript lists were used for gene ontology (GO) enrichment analysis. GO term enrichment (biological process) was analyzed using the PANTHER tool (Mi et al., 2017). The list of differentially expressed genes was analyzed separately.

The lists of upregulated or downregulated transcripts (fold change ≥ 2 or ≤−2) were uploaded to the online software STRING 10.5^3^ database and analyzed using default parameters without expansion. In addition, enrichment results for KEGG pathways, GO, and InterPro were also obtained from STRING, using the false discovery rate (FDR) for multiple testing correction (*p* < 0.05).

The obtained protein-protein interaction networks were downloaded and analyzed using Cytoscape 3.6.1 software (Shannon, 2003). The binary networks obtained from this screen were analyzed with the Molecular Complex Detection (MCODE) plugin to identify subnetworks with scores ≥ 2.5 (Bader and Hogue, 2003). Centrality parameters (node degree and betweenness) of each node were analyzed using the cytoHubba plugin (Chin et al., 2014). Biological process categories were generated by functional enrichment for a given cluster and category, with significance (*p*) assessed as a hypergeometric distribution. Multiple tests were also corrected using an FDR algorithm, which was fully implemented in the BiNGO software, with a significance level of *p* < 0.05 (Rivals et al., 2007).

The potential master regulators (TFs) of downregulated and upregulated networks were predicted using the iRegulon plugin version 1.3. The criteria for motif enrichment analysis were set as the identity between orthologous genes = 0.05, and a maximum FDR of motif similarity = 0.001. The consensus was searched in sequences up to 10 K in the promoter region using *Homo sapiens* as the reference. The motif with a normalized enrichment score (NES) of ≥4 was set as the threshold (Verfaillie et al., 2015).

### Quantitative PCR

The qPCR reactions were prepared using 2Power SYBR Green PCR Master Mix (Life Technologies) and proceeded on an Applied Biosystems Real-Time PCR system. Briefly, PCR was performed using specific primers, 1 × Quantifast SYBR Green PCR master mix, and 10 ng of template cDNA in a 10 μL reaction volume. Reaction mixtures were initially denatured at 95°C for 5 min, followed by 45 cycles of 60°C for 1 min with a final melt at 45°C for real-time PCR analysis. Each assay was replicated using three independent biological samples. Cycle threshold (C_t_) values were averaged for target genes and normalized against GAPDH (endogenous reference gene), and gene expression was quantified using the 2^–ΔΔCT^ method. We validated the RNA-Seq data using the following equation: ΔΔC_t_ inhibitor = ΔC_t_ inhibitor – ΔC_t_ LPS. All primers were quality controlled to ensure that each set (forward and reverse) generated a specific PCR product. Primer’s information has been provided in Supplementary Table 1.

### Western Blotting

The expression of selected proteins in U937 cells was investigated by western blotting. Total protein was extracted using IllustratriplePrep Kits (GE Healthcare), as described by the manufacturer. Lysates (20 μg) were separated on sodium dodecyl sulfate (SDS)-polyacrylamide gels and transferred to PVDF membranes, as described by Laemmli (1970). Membranes were incubated in a blocking buffer (5% dried milk, 0.5% Tween-20 in TBS, pH 7.2) for at least 40 min, then overnight at 4°C with the following primary antibodies: against APE1, NRF1, β-actin, and ELK1 (Santa Cruz Biotechnology) (1:1,000), MYC (Abcam) (1:1,000), and NF-κB p65 subunit (1:1,000; Millipore). Further, the blots were washed with TBST and incubated with a horseradish peroxidase-conjugated secondary antibody (1:1,000; Santa Cruz Biotechnology) for 1 h at room temperature. They were then immersed in Amersham ECL Prime western blotting Detection Reagent (GE Healthcare) before imaging on a Chemidoc System (Bio-Rad). Data are shown as those from three independent experiments. Expression levels were estimated using β-actin as the loading control in ImageLab software.

### Microfluidic Gel Electrophoresis of Ribosomal RNA

The RNA integrity of U937 cells was analyzed using a chip-based microcapillary electrophoresis system (Agilent 2100 BioAnalyzer; Agilent Technology) and Agilent RNA 6000 Nano Chips. The reference was the RNA molecular weight ladder provided in the kit. The samples were resolved by electrophoresis, as described by the manufacturer. The molecular weight and integrity of rRNA were determined by the ratio of 28S/18S peaks using Agilent 2100 Expert Software.

### Cell Cycle Analysis by Flow Cytometry

The DNA content and cell cycle distribution in U937 cells were determined by flow cytometry. The cells were harvested and pelleted by centrifugation, washed twice in phosphate-buffered saline (PBS, pH 7.2), and fixed with 70% cold absolute ethanol for at least 12 h at 4°C. Immediately before cell cycle determination, the cells were gently resuspended and stained with propidium iodide (PI; 20 μg/mL) and 10 μg/mL RNAse (Sigma-Aldrich) in PBS) and incubated at 37°C for 60 min. We acquired 10,000 events per sample using a flow cytometer (Becton Dickinson and Co., Franklin Lakes, NJ, United States) and a 488 nm argon laser. The data were analyzed using FlowJo 7.6.5 software (FlowJo LLC., Ashland, OR, United States).

### Statistical Analysis

The normality of the data was assessed using the Kolmogorov-Smirnov test. Groups were evaluated using two-way analyses of variance (ANOVA), and individual groups were analyzed by Student’s *t*-test using GraphPad Prism 5 (GraphPad Software, San Diego, CA, United States). Data are expressed as the mean ± SE. Values with *p* < 0.05 were considered statistically significant.

## Results

### Methoxyamine or E3330 Treatment Does Not Alter the Viability of U937 Cells After 24 h or the Expression of APE1/Ref-1 During Inflammation but Decreases the Expression of Proinflammatory Cytokines

**FIGURE 1:**
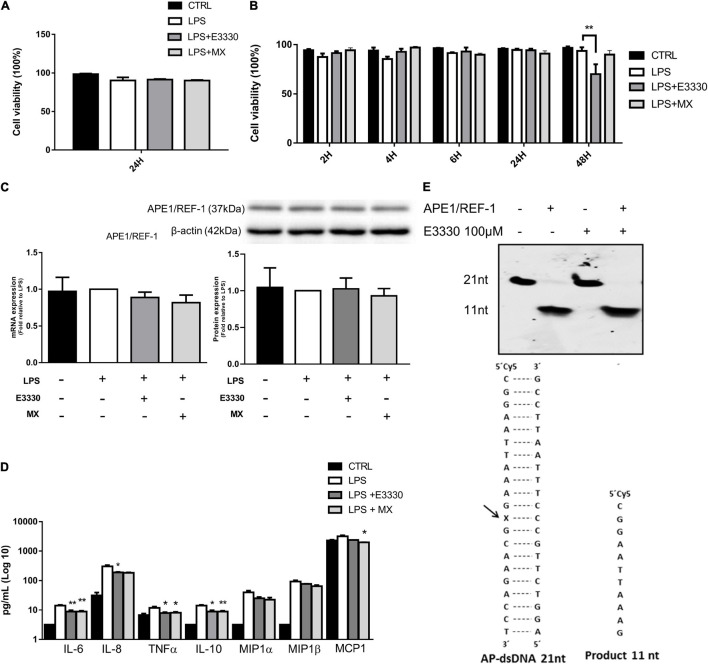
Effect of E3330 and methoxyamine on apyrimidinic endonuclease 1/redox factor-1 (APE1/Ref-1) and inflammatory response in U937 cells. **(A)** Cell viability measurement after lipopolysaccharide (LPS) (1 μg/mL) stimulation for 24 h followed by treatment with E3330 (100 μM) or MX (6 mM) for 4 h. **(B)** Cell viability after 1 h of LPS stimulation and subsequent E3330 or MX treatment for 2, 4, 6, and 24 h. Two-way analyses of variance (ANOVA) was used to compare the treatment groups. **(C)** mRNA and APE1/Ref-1 protein expression after LPS stimulation for 24 h followed by treatment with E3330 or methoxyamine (MX) for 4 h. **(D)** Protein levels of cytokines and chemokines after LPS 24 h plus E3330 or MX for 4 h. Unpaired Student’s *t*-test was performed for inflammatory response measurement. *p* < 0.05 was considered statistically significant. **(E)** Cleavage electrophoretic profiles of oligonucleotides (left) and double-stranded oligonucleotides containing an abasic site and cleavage product (right) E3330 did not alter APE1 endonuclease activity. *p < 0.05, **p < 0.01.

Stimulation for 24 h with LPS (1 μg/mL) followed by incubation with E3330 (100 μM) or methoxyamine (MX) (6 mM) did not significantly affect the viability of U937 cells (Figure 1A). Similar data were obtained after stimulation for 1 h with LPS, followed by inhibitor addition. Until 24 h of exposure, no significant alterations in monocyte viability were observed compared to non-stimulated cells. However, after 48 h of treatment with LPS + E3330, we observed a significant decrease in the viability of U937 cells compared to that of LPS-stimulated cells (Figure 1B).

Inhibition of APE1/Ref-1 activities decreased the expression of proinflammatory cytokines and chemokines in response to LPS treatment, indicating that AP site repair and the redox function of APE1/Ref-1 are vital for expressing these genes (Figure 1C). Owing to its ability to self-regulate, we analyzed APE1/Ref-1 expression after treatment. We observed no changes in APE1/Ref-1 protein or mRNA levels (Figure 1D). In addition, using *in vitro* repair assays, we also observed that E3330 did not affect DNA repair endonuclease activity (Figure 1E).

### Methoxyamine and E3330 Alter the Expression of Genes Related to Inflammatory Response, Mitochondrial Gene Expression, and rRNA Metabolism

**FIGURE 2:**
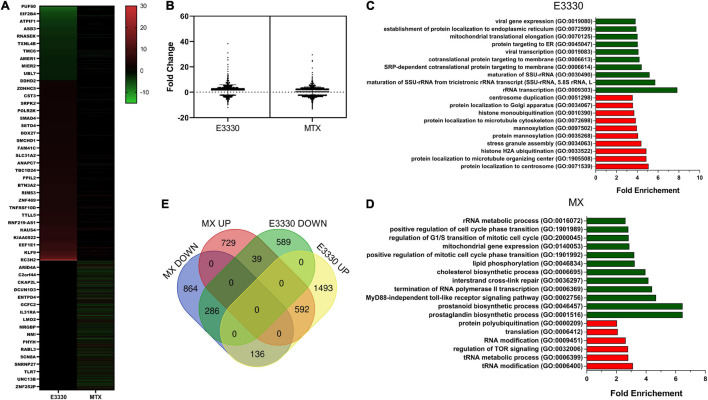
RNA-seq data analysis. **(A)** Heatmap of differentially expressed genes after MX or E3330 treatment during inflammatory stimulation with LPS. **(B)** Distribution of differentially expressed genes according to the fold change. **(C)** Venn diagram describing the number of differentially expressed genes after MX and E3330 treatments. **(D)** Biological processes enriched between upregulated (red) and downregulated (green) genes after E3330 treatment. **(E)** Biological processes enriched between upregulated (red) and downregulated (green) genes after MX treatment. Gene ontology (GO) analysis was performed using the Panther classification system. All processes described were significant (*p* < 0.05).

Transcriptome analysis was used to investigate the profile of transcriptional changes in response to the inhibition of AP site repair or redox activity of APE1/Ref-1 and their involvement in inflammatory modulation. Comparative transcriptome analysis of U937 cells stimulated with LPS + E3330 and LPS-stimulated cells revealed 914 downregulated (fold change ≤ −2) and 2,222 upregulated genes (fold change ≥ 2). MX addition after LPS stimulation induced downregulation of 1,287 genes and upregulation of 1,362 genes (Figure 2). Sequencing results were validated using qPCR. For validation, six genes were chosen among the downregulated and upregulated genes in both treatments. The results shown in Supplementary Figure 1 demonstrate that all genes evaluated presented similar expression patterns, both in RNA sequencing and qPCR analysis.

The list of upregulated and downregulated genes (fold change ≥ 2 or ≤−2) was submitted for GO evaluation in Panther. The most enriched biological processes for each gene list are represented in Figures 2C,D. Regarding downregulated genes, we observed enrichment of genes related to mitochondrial gene expression and rRNA metabolic process after both treatments. Moreover, MX treatment also decreased gene expression related to the prostaglandin biosynthetic process (e.g., PTGES3, PTGES2, and PTGS1/COX-1) and MyD88-independent toll-like receptor signaling pathway (e.g., TRAF2, IKBKG, and TLR4). Together with the inhibition of cytokine expression, these results indicate a negative regulation of genes related to the inflammatory process after inhibition of AP site repair.

We constructed a Venn diagram with four sets of genes (Figure 2E). The results showed that 286 genes were negatively regulated, while 592 genes were positively regulated after both treatments.

### E3330 Increases the Coupling of Apurinic-Apyrimidinic Endonuclease 1/Redox Factor-1 to the TNF4 Promoter and Decreases the mRNA Expression

**FIGURE 3:**
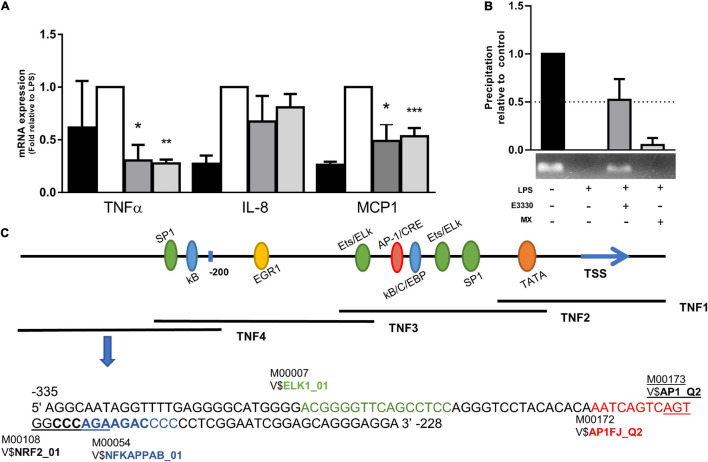
E3330 treatment changes APE1/REF-1 DNA occupancy in TNF4 promoter. **(A)** mRNA levels of TNF-α and MCP1 were reduced by E3330 and MX treatment after LPS stimulation. Unpaired Student’s t-test was performed for inflammatory response measurement. *p* < 0.05 was considered statistically significant. **(B)** PCR of TNF4 promoter after APE1/Ref-1-Ab-ChIP revealed that APE1/Ref-1 coupling is reduced after treatment with LPS compared to the control. E3330 increases APE1/REF-1 DNA occupancy in TNF4 promoter. **(C)** Representative scheme of the TNF-α promoter with the representation of transcription factors that bind to it. Highlighted region in TNF4 with binding motifs predicted by research in the Transfac database. *p < 0.05, **p < 0.01, ***p < 0.001.

Analyses of mRNA levels showed that both inhibitors act in the transcriptional regulation of cytokines such as TNF-α and MCP1 (Figure 3A). To investigate the potential implication of APE1/Ref-1 repair activity in the inflammatory response, we evaluated whether the inhibition of AP sites by MX in the TNF4 promoter (of TNF-α) guanine-rich sequence would be different from that found in E3330. PCR amplification of the TNF4 promoter segment after APE1/Ref-1-Ab-ChIP DNA demonstrated that APE1/Ref-1 coupling was drastically reduced after LPS treatment compared to the control. However, the redox inhibition of APE1/Ref-1 increased the coupling of APE1/Ref-1 in the TNF promoter (Figure 3B). Interestingly, this result corroborates the findings of immunofluorescence, in which U937 cells without any treatment showed the APE1/Ref-1 protein located mainly in the nucleus, and after LPS stimulation, a marked increase in the cytoplasm was observed. In both E3330 and MX treatments, APE1/Ref-1 reduced its cytoplasmic location and was translocated to the nucleus (Supplementary Figure 2). To investigate the coupling of APE1/Ref-1 in the TNF4 promoter, we searched for transcription factor binding motifs in this region. Using TRANSFAC software, we identified motifs for ELK1, AP1, NRF2, and NF-κB (Figure 3C).

### Nuclear Respiratory Factor 1 and the ETS Family of Transcription Factors Were the Master Regulators of the Negatively Expressed Genes

**FIGURE 4:**
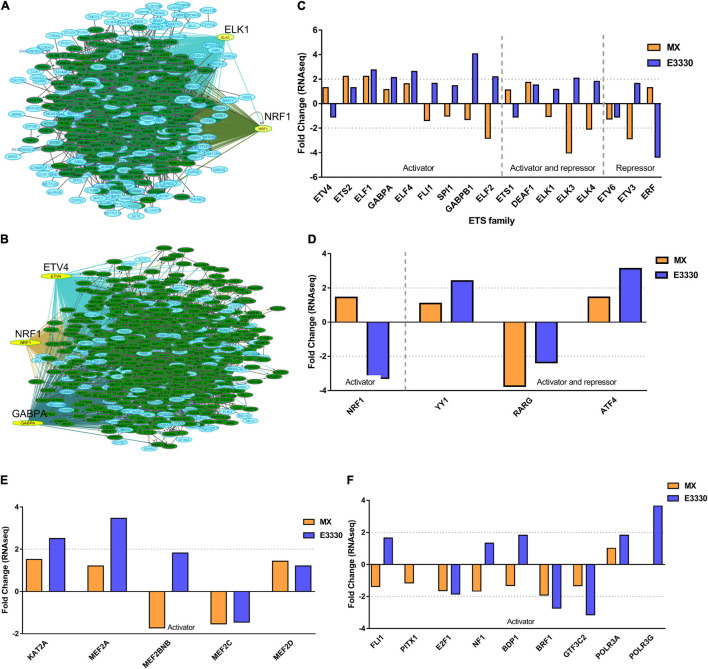
RNAseq data of critical transcription factors predicted by iRegulon in response to exposure to inhibitors. Protein-protein interaction (PPI) networks generated by STRING formed by the products of differentially regulated genes were analyzed by Cytoscape and its master regulators predicted by the iRegulon plugin. Mater regulators predicted by iRegulon are highlighted in yellow, targets of the master regulators are shown in green. In blue, genes are not targets of the master regulators. **(A)** E3330 downregulated network. **(B)** MX downregulated network. **(C)** ETS family transcription factors found in RNAseq. TFs were divided into transcription activators and repressors or both, according to the Protein Atlas database. **(D)** NRF1, RARG, YY1, and ATF4 master regulators of at least one of the downregulated networks. **(E)** KAT2A and MEF2A master regulators of E3330 upregulated network compared to MX treatment. **(F)** FLI1, BDP1, and PITX1 master regulators of MX upregulated network compared to the E3330 treatment.

To identify the central genes and regulators of differentially expressed gene lists, we built a protein–protein interaction (PPI) network (Figures 4A,B and Supplementary Figure 3). All PPI networks were subjected to a centrality analysis (Supplementary Figure 4) and then analyzed by iRegulon to predict enriched motifs and their master regulators. After treatment with MX and E3330, iRegulon identified 34 TFs capable of binding to motifs represented in Tables 1, 2, most of which belong to the ETS family. Fourteen TFs were not expressed in U937 cells according to the Protein Atlas database, and were not detected in our RNAseq. The others were considered transcription activators (*n* = 9), repressors (*n* = 5), or with both actions (*n* = 3) using the Protein Atlas (Figure 4C). From the downregulated network after E3330 treatment, ELK1 motifs were predicted to be the most significant. In contrast, ETV4 and GABPA were most likely in the MX-downregulated network.

**TABLE 1:** Description of master regulators of differentially expressed genes in response to E3330 treatment predicted by iRegulon.

Transcription factors	Motifs	NES	Targets number
**LPS + E3330 down-regulated network**			
ELK1	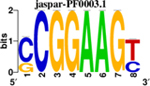	5.802	457
NRF1	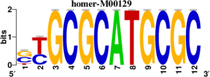	4.521	315
**LPS + E3330 upregulated network**			
YY1	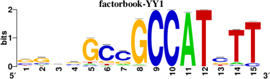	3.672	221
KAT2A	^*^	3.438	82
MEF2A	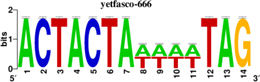	3.241	630

^*^This motif could be shown by iRegulon, as it is part of TRANSFAC pro.

**TABLE 2:** Description of master regulators of differentially expressed genes in response to methoxyamine (MX) treatment predicted by iRegulon.

Transcription factors	Motifs	NES	Targets number
**LPS + MX downregulated network**			
ETV4	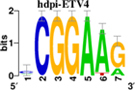	6.451	602
GABPA	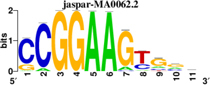	5.893	629
NRF1	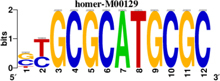	5.021	396
YY1	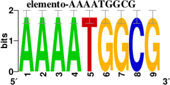	4.357	126
**LPS + MX upregulated network**			
CRX	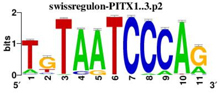	5.125	386
FLI1	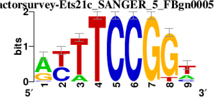	4.616	688
BDP1	^*^	4.033	259

^*^This motif could shown by iRegulon, as it is part of TRANSFAC pro.

Some differences in RNAseq expression were noted, such as the downregulation of some members of the TCF subfamily (ELK3 and ELk4) and SRF after treatment with MX (fold change = −4.02, −2.08, −2.62, respectively). On the other hand, the ERF repressor was downregulated (−4.37) after E3330 treatment and upregulation of several ETS such as GABPA (2.13), ELF1 (2.74), GABPAB1 (4.06), ELF4 (2.62), and ELF2 (2.19) (Figure 4C) was observed. Furthermore, enrichment of ETS binding motifs among MX-upregulated genes was also observed. FLI1 was the most likely transcription factor involved in the regulation of these genes. The list of likely TFs predicted by iRegulon is presented in Tables 1, 2.

Similarly, 396 downregulated genes after MX treatment and 315 downregulated genes after E3330 showed binding motifs to the NRF1 transcription factor (Tables 1, 2). These data indicate that the inhibition of APE1/Ref-1 can directly or indirectly regulate the expression of NRF1 targets. Also, YY1/YY2 motifs in genes negatively regulated for MX and positively regulated by E3330 were observed. We also submitted to iRegulon for each set of genes resulting from the Venn diagram. This result showed that NRF1, ELK1, and GABPA motifs-maintained enrichment among genes downregulated by both inhibitors (Supplementary Figure 5). The set of genes downregulated exclusively upon MX or E3330 treatment had binding motifs to RARG and ATF4, respectively. In addition, RARG (−3,74) expression decreased after MX treatment (Figure 4D), while YY1, ATF4, KAT2A, MEF2A, and POLR3G increased expression after E3330 treatment (Figures 4D–F).

### Methoxyamine and E3330 Decrease the Expression of Nuclear Respiratory Factor 1 Targets Related to Mitochondrial Organization

**FIGURE 5:**
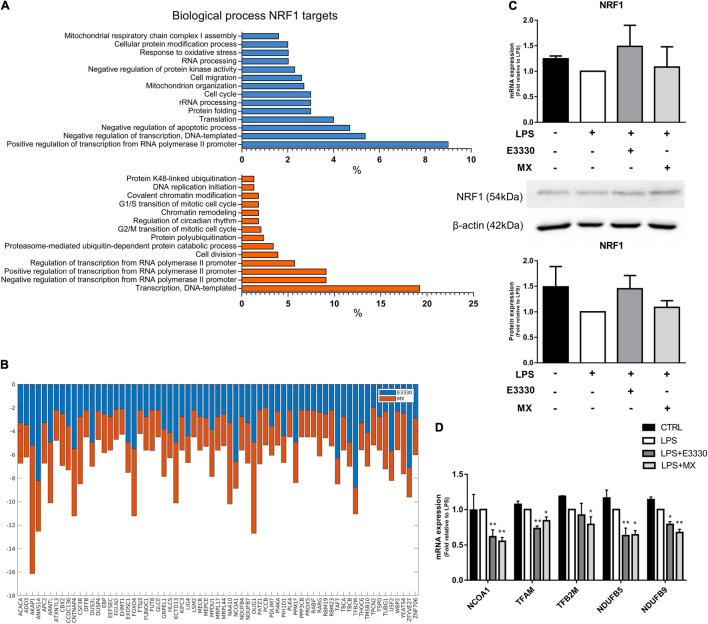
E3330 and MX treatment decreased the expression of nuclear respiratory factor 1 (NRF1) targets in U937 cells during LPS stimulation. **(A)** Biological process enriched between NRF1 targets downregulated after E3330 treatment (blue) and MX treatment (orange). All processes described were significant (*p* < 0.05). **(B)** Expression of NRF1 targets common to both treatments in RNAseq. **(C)** Protein and mRNA NRF1 expression analyzed by western blot and q-PCR in U937 cells. **(D)** mRNA expression of NRF1 target genes upon LPS, MX, and E3330 treatment. Unpaired Student’s *t*-test was performed and *p* < 0.05 was considered statistically significant. *p < 0.05, **p < 0.01.

Nuclear Respiratory Factor 1 has many targets in both networks. The inhibition of APE1/Ref-1 redox activity downregulated 315 NRF1 targets related to several biological processes, such as regulation of transcription, mitochondrion organization, translation, and response to oxidative stress (Figure 5A, blue). In contrast, MX treatment downregulated 396 targets related to transcription, protein polyubiquitination, chromatin remodeling, and the cell cycle (Figure 5A). Some genes, such as *TFB2M* and *NCOA1*, are common to both treatments and are shown in Figure 5B. However, we observed that inhibitors did not significantly change NRF1 protein or mRNA expression (Figure 5C), indicating that APE1/Ref-1 regulates NRF1 activity and not its expression. We selected and analyzed, using qPCR, the expression of commons and exclusive genes downregulated by MX or E3330 and confirmed a decrease in the expression of NRF1 targets, corroborating the data obtained by iRegulon. Furthermore, AP repair inhibition promoted a significant reduction in mitochondrial gene expression, such as *TFAM*, *TFB2M*, *NDUFB5*, and *NDUFB9*. Similarly, the redox inhibition of APE1/Ref-1 also decreased the expression of the same genes (except for *TFB2M*), indicating that both inhibitors can act in the transcriptional regulation of these genes (Figure 5D).

### Transcription Factors of the ETS Family Were Associated With the Expression of Genes Related to Transcription and rRNA Metabolism

**FIGURE 6:**
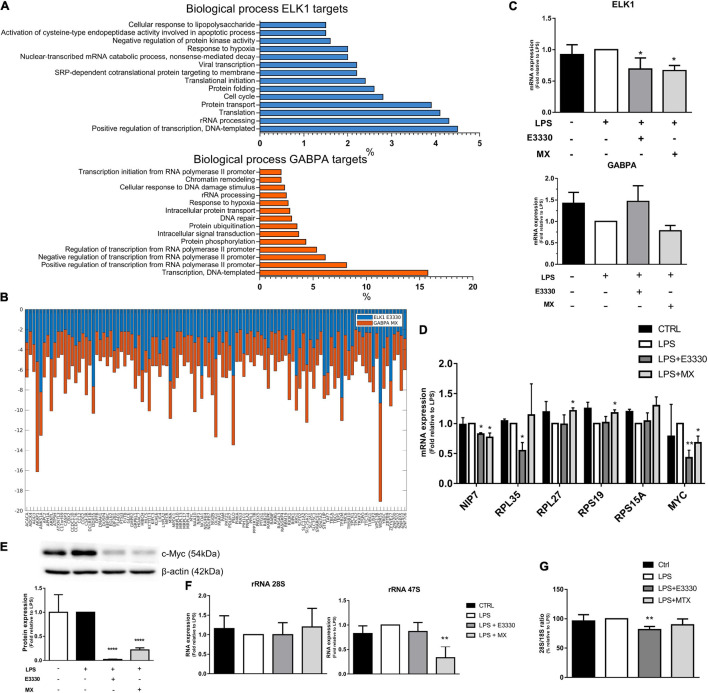
The ELK1 and GABPA targets related to ribosomal biogenesis were regulated differently after E3330 and MX treatment. **(A)** Biological process enriched between downregulated ELK1 targets genes after E3330 treatment (blue) and downregulated GABPA targets genes after MX treatment (orange). All processes described were significant (*p* < 0.05). **(B)** Expression of ELK1 and GABPA targets common to both treatments. **(C)** mRNA expression of ELK1 and GABPA analyzed by qPCR in U937 cells. **(D)** mRNA expression of genes related to ribosomal biogenesis upon LPS, MX, and E3330 treatment. **(E)** MYC protein expression was analyzed by western blot. **(F)** Ratio 28S/18S and expression of rRNA after MX and E3330 treatment. Unpaired Student’s *t*-test was performed and *p* < 0.05 was considered statistically significant. *p < 0.05, **p < 0.01, ***p < 0.001, ****p < 0.0001.

Transcription factors belonging to the ETS family, such as ELK1, GABPA, and ETV4, were also identified by iRegulon as being responsible for the expression of most genes downregulated for APE1/Ref-1 inhibition; among them, the ELK1 motifs were enriched among the genes downregulated in the E3330 treatment. In comparison, ETV4 and GABPA were enriched among the genes downregulated in the MX treatment. Interestingly, ELK1 targets were related to rRNA processing and translation, mainly in the E3330 network (Figure 6A).

The ETS family has significant redundancy among its binding motifs; therefore, several targets were also common between ELK1 and GABPA (Figure 6B). Although some genes related to rRNA metabolism were also downregulated upon MX treatment, and ELK1 mRNA expression was decreased after both treatments (Figure 6C), the selected targets for qPCR analysis showed different expression regulation among inhibitors. For example, E3330 treatment decreased the expression of *RPL35*, *MYC*, and *NIP7*. In contrast, repair inhibition of AP sites by MX increased the expression of some ribosomal proteins (RPS19 and RPL27) and decreased MYC protein and mRNA expression (Figures 6D,E). In addition, the expression of rRNA 47S was significantly reduced by MX, indicating that the repair of AP sites affects rRNA transcription (Figure 6F). On the other hand, the 28S/18S ratio decreased significantly after E3330 treatment, indicating that APE1/Ref-1 redox activity is essential for rRNA processing (Figure 6G).

### Inhibition of Apyrimidinic Endonuclease 1/Redox Factor-1 Redox Function Affects the Control of Cell Growth and Stress Response

**FIGURE 7:**
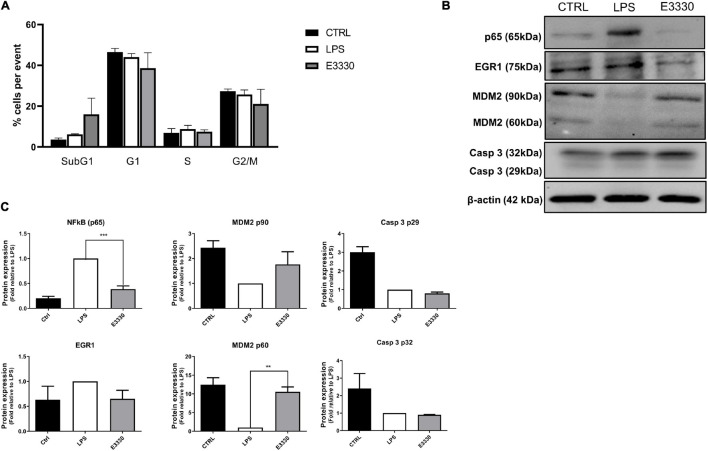
Inhibition of APE1/Ref-1 redox function affects the cell growth and expression of different proteins related to stress response. **(A)** Cell cycle assays after stimulation with LPS for 24 h followed by incubation with E3330 for 4 h show a discrete increase ratio (%) of subG1cells. **(B)** Representative western blot analysis of whole-cell extracts and **(C)** histogram reporting densitometric quantification of western blotting signals from at least three independent experiments. β-actin was used as the loading control. Unpaired Student’s *t*-test was performed and *p* < 0.05 was considered statistically significant. **p < 0.01, ***p < 0.001.

Cell viability was not affected after 24 h of LPS stimulation and treatment with E3330 or MX (Figure 1B). However, a reduction of viable cells at the time point of 48 h of treatment with E3330 was observed, and flow cytometry analysis revealed a slight increase in the ratio of subG1 cells (Figure 7A), indicating a role for APE1/REF-1 redox regulation in the control of cell growth. Furthermore, we could observe that p65 (RELA) reduced expression at the protein level after E3330 treatment (Figures 7B,C), corroborating the transcriptome data. A statistically significant increase in the expression of MDM2 (60 kDa cleaved portion) was also observed after E3330 treatment. However, no significant difference in the protein expression of EGR1 and Casp3 was observed (Figures 7B,C). These data corroborate our findings for the upregulated networks.

## Discussion

Our data revealed the downregulation of several transcriptional regulators and immune response-activating signal transduction genes for both E3330 and MX treatments. These data indicate that APE1/Ref-1 acts on the transcriptional regulation of cytokines and inflammatory modulators during LPS signaling. NF-κB is a crucial transcriptional factor involved in the inflammatory process and is a redox APE1/Ref-1 target; its DNA-binding activity is impaired when APE1/Ref-1 redox function is inhibited (Mitomo et al., 1994; Hiramoto et al., 1998). In this study, we observed the downregulation of p65 (RELA gene), the catalytic subunit of NF-kB, after APE1/Ref-1 redox inhibition by E3330. Therefore, inhibiting APE1/Ref-1 redox activity might decrease inflammatory modulators’ expression by inhibiting NF-κB binding in gene promoters and reducing the expression of its p65 catalytic subunit in U937 cells.

We also showed a new close relationship between APE1/Ref-1 and a region of the TNF promoter, called TNF4. Interestingly, APE1/Ref-1 was found attached to TNF4, and reduced levels of TNF-α were observed after E3330 treatment in LPS-stimulated cells, suggesting that APE1/Ref-1 redox activity is associated with repression of TNF4 in U937 cells. Furthermore, this promoter region contains an ELK1 binding site, which can act as a corepressor linked to complexes with HDAC-1 and LSD1 (Yang et al., 2001; Gerosa et al., 2020), both of which are APE1/Ref-1 partners (Bhakat et al., 2003; Amente et al., 2010). Conversely, MX treatment did not promote APE1/Ref-1 attachment on TNF4, but reduced expression of TNF-α were observed.

In cells stimulated with TNF-α, the binding of OGG1 to 8-oxoG in regions close to the TNF-α promoter increased the DNA occupancy of NF-κB and gene expression via epigenetic regulation (Pan et al., 2016). Furthermore, we also observed a decrease in TNF-α and MCP1 mRNA expression during impairment of endonuclease activity by MX. These results indicate that the resolution of 8-oxoG, more specifically AP sites, is crucial for cytokine expression.

In this study, we have demonstrated that both inhibitors decreased the expression of genes related to mitochondrial gene expression and rRNA metabolic process in LPS-stimulated monocytes (Figures 3C,D). In addition, these genes showed enrichment of binding motifs to the transcriptional factor NRF1 (Figure 4). NRF1 is one of the main regulatory factors of mitochondrial biogenesis, often referred to as a transcription activator (Gleyzer et al., 2005; Piantadosi and Suliman, 2012). In addition, chip-on-chip and chip-seq studies have revealed that NRF1 binds to genes associated with RNA metabolism, DNA damage repair, chromosome organization, and cell cycle (Cam et al., 2004; Satoh et al., 2013; Bhawe and Roy, 2018). It has already been observed that the lack of aprataxin leads to reduced levels of APE1/Ref-1, which in turn is related to the reduction of NRF1 levels and consequent mitochondrial dysfunction (Garcia-Diaz et al., 2015). Furthermore, APE1/Ref-1 redox function is involved in controlling the DNA-binding activity of NRF1. In the absence of APE1/Ref-1 redox function, the expression of NRF1 target genes was significantly reduced (Li et al., 2012).

Amente et al. (2010) observed that LSD1 produces H_2_O_2_, increasing the oxidation of guanines in MYC target gene promoters. The presence of 8-oxoG in DNA recruited OGG1 and APE1/Ref-1 and improved gene expression (Amente et al., 2010). LSD1 is also a member of the transcriptional corepressor complex CoREST, a unique complex containing both a histone demethylase (LSD1) and a deacetylase enzyme (HDAC1) (Song et al., 2020). The association between the NRF1 motif and LSD1 occupancy has been reported in different cell lines (Benner et al., 2013). Hence, blockage of AP sites by MX during gene regulation may be the reason for the decrease in NRF1 targets after MX treatment. This hypothesis should be tested in future studies.

Here, we also identified a consensus signature for the ETS family of TFs, which have 28 members in the human genome and significant redundancy among their binding motifs; consequently, diverse targets were also identified among several regulators (Sizemore et al., 2017). Among ETS factors, GABPA and ELK1 were shown to be master regulators of downregulated genes. Both GABPA and ELK1 exhibit target redundancy and control the same biological processes, including ribosome biogenesis, mitochondrial processes, cytoskeleton, and cell migration. However, despite the redundancy of targets and functions, these regulators also present a cohort of specific target genes (Boros et al., 2009; Odrowaz and Sharrocks, 2012).

Although a set of ETS target genes was found to be mainly downregulated upon MX inhibition, ELK1 mRNA expression was decreased after both treatments (Figures 6C,D). Furthermore, we noted that the biological processes involved in ribosomal biogenesis were more representative of the E3330 transcriptome. Interestingly, ELK1 targets were related to rRNA processing and ribosomal biogenesis, mainly in the E3330 network (Figure 6A). In addition, the 28S/18S ratio was significantly lower in cells treated with E3330, suggesting inefficient rRNA processing. These data indicate that the redox inhibition of APE1/Ref-1 is more effective in regulating rRNA processing. In contrast, MX treatment decreased the expression of rRNA 47S without affecting rRNA processing.

ETS transcription factors are generally activated by phosphorylation and binding in specific sequences such as RAS-responsive elements (RREs) and, in TCF subfamily cases, serum response elements (SRE). ETS-binding sequences act as RREs when flanked by AP-1 binding sites, and enhancer activation requires ETS1 and AP-1 activation (Wasylyk et al., 1998; Yordy and Muise-Helmericks, 2000; Hollenhorst et al., 2011). It is known that the redox activity of APE1/Ref-1 facilitates AP-1 DNA binding and activity (Xanthoudakis and Curran, 1992; Ando et al., 2008). Therefore, E3330 treatment can decrease AP-1 activation and disturb the expression of genes that have RREs. In contrast, we observed that MX treatment decreased the expression of the TCF subfamily (ELK1, ELK4, and ELK3), including SRF. Thus, these TFs can act as transcription activators and repressors that bind to the SRE (For review, Yordy and Muise-Helmericks, 2000; Shaw and Saxton, 2003; Buchwalter et al., 2004). We observed an enrichment of ETS motifs among MX-upregulated genes, indicating activation of transcription activators or the absence of a repressor.

Several pathways play an essential role in response to LPS stimulation. The ERK pathway is responsible for the phosphorylation of TFs such as ELK-1 and FLI1, leading to their activation and consequent induction of genes related to inflammatory response, differentiation, and cell growth (Guha and Mackman, 2001). Furthermore, changes in the redox state of ERK proteins are associated with their activation and inhibition (Keyes et al., 2017). For example, it was demonstrated that APE1/Ref-1 forms a complex with ERK2 and rescues ERK oxidative inactivation through its redox function, favoring cyclin D1 expression and cell cycle progression G1-to-S passage (Wang et al., 2013). Thus, in our model, E3330 treatment can compromise cellular responses dependent on the ERK pathway, which was not observed in the MX treatment.

We should also consider TFs that were not enriched by iRegulon analysis but are classic APE1/Ref-1 redox activity targets; examples include EGR1 and Jun/Fos (AP-1) (Xanthoudakis and Curran, 1992; Huang and Adamson, 1993; Pines et al., 2005; Ando et al., 2008; Fantini et al., 2008). Binding sites to the SP family transcription factor were found to be enriched exclusively in E3330 downregulated genes (represented by SP8; Supplementary Figure 5), which has an overlap of targets with EGR1. Similarly, genes such as *RPL35*, *ESRRA*, and *RelA*, downregulated by E3330 treatment in monocytes, also have binding sites to EGR1 and Jun. In addition, EGR1 is a known activator of the ELK1 gene promoter in monocytes (Lehmann et al., 1999). In addition, we also observed decreased expression of ELK1 after MX treatment, which can be associated to the presence of CpG-rich regions in the ELK1 gene promoter that targets active demethylation by TED enzymes (Qu et al., 2017), suggesting that APE1/Ref-1 may also be related to DNA repair-dependent ELK1 expression control. These results indicate that the redox and repair activities of APE1/Ref-1 can regulate gene expression through independent but overlapping mechanisms.

In addition, in the iRegulon analysis, we observed enrichment of binding sites for RARgamma (RARG) into downregulated genes exclusively after MX treatment. RARG is a nuclear retinoic acid receptor (RAR) that forms heterodimers with RXRs. The redox function of APE1/Ref-1 mediates the binding of RARs to retinoic acid-responsive elements (RARE) (Robertson et al., 2006; Fishel et al., 2010). RAR and estrogen receptor (ER) have overlapping DNA-binding sites and may act cooperatively or antagonistically (Liu et al., 2014). The RAR and ER pathways control cell differentiation, stress response, and immune homeostasis (Straub, 2007; Oliveira et al., 2018). In our study, we observed downregulation of RARG after both treatments. However, ESRRA (an estrogen receptor member) is upregulated in MX and downregulated after E3330 treatment. Estrogen and retinoic acid-responsive gene promoters are DNA oxidation targets mediated by LSD1, which recruits BER enzymes, favoring chromatin remodeling (Perillo et al., 2008; Zuchegna et al., 2014).

Binding sites for YY1/YY2 were also enriched between MX-downregulated and E3330 upregulated genes. YY1 and YY2 are homologous proteins that show overlapping DNA binding sites and can act as synergistic or antagonistic activators or repressors, and are involved in regulating cellular processes such as inflammation, stress response, and cell cycle control (Klar and Bode, 2005; Chen et al., 2010; Li et al., 2020). Li et al. (2014) showed the direct repression of CBF/NF-Y/YY1 DNA-binding activities by E3330, suggesting that YY1 is an APE1 target.

Several studies have revealed a connection between DNA damage response, DNA repair, and rRNA metabolism pathways (Larsen and Stucki, 2016; Vohhodina et al., 2016). In the E3330 treatment, we observed the upregulation of several genes involved in cell cycle control, DNA damage response, and DNA repair, such as PIK3CA, CDK1, and ATR (Supplementary Figure 4), which are classified as hub-bottlenecks, as well as an increase in the expression of MDM2 when compared to LPS (Figure 7). MDM2 is a stress sensor, and MDM2-mediated ubiquitination can signal APE1/REF-1 degradation following treatment with genotoxicants (Busso et al., 2009). APE1/REF-1 redox inhibition seems to induce cell stress higher than the inhibition of DNA repair activity in our experimental model.

In summary, the selective inhibition of APE1/Ref-1 can alter several cellular processes and understanding the mechanism underlying protein regulation would be a valuable target for both preventative and curative treatment paradigms. Furthermore, the molecular mechanisms responsible for the various functions of APE1/Ref-1 need to be elucidated to develop more targeted therapies for a wide range of human diseases. Our data showed that the AP site repair and redox functions of APE1/Ref-1 are essential for modulating genes related to the global inflammatory response through direct and indirect pathways. In addition, redox and repair activities are also necessary for the transcription of genes related to basal transcription, cell cycle, ribosomal biogenesis, and mitochondrial biogenesis, suggesting that both functions affect transcriptional regulation by different but overlapping mechanisms, thus, indicating that these functions are not entirely independent, as initially proposed. Finally, this work indicates several new TFs that may be APE1/Ref-1 function targets.

## Data Availability Statement

The datasets presented in this study can be found in online repositories. The names of the repository/repositories and accession number(s) can be found below: NCBI GEO, accession no: GSE182813.

## Author Contributions

TO, FF-D, RM, DP, and LC developed the experimental assays. TO, FF-D, VS, and SS performed RNAseq data analysis. LA-L, TO, and FF-D conceived the study and its design, performed data analysis, and drafted the manuscript. The final manuscript was reviewed and authorized by all authors.

## Conflict of Interest

The authors declare that the research was conducted in the absence of any commercial or financial relationships that could be construed as a potential conflict of interest.

## Publisher’s Note

All claims expressed in this article are solely those of the authors and do not necessarily represent those of their affiliated organizations, or those of the publisher, the editors and the reviewers. Any product that may be evaluated in this article, or claim that may be made by its manufacturer, is not guaranteed or endorsed by the publisher.

## Footnotes

1
http://hgdownload.soe.ucsc.edu/goldenPath/hg19/bigZips/refMrna.fa.gz

2
http://hgdownload.cse.ucsc.edu/admin/exe/

3
https://string-db.org/
