# Supplementary material for: Chemical Inhibition of Apurinic-Apyrimidinic Endonuclease 1 Redox and DNA Repair Functions Affects the Inflammatory Response via Different but Overlapping Mechanisms
Source: Front Cell Dev Biol. 2021 Sep 20;9:731588. doi: 10.3389/fcell.2021.731588 (PMC8488223; doi:10.3389/fcell.2021.731588)
Supplement: Supplementary file 3 [file Presentation_1.pdf]

Supplementary table 1. List of primers for q-PCR analisys in U937 cells.

Gene	Primer Forward	Primer Reverse
**GAPDH**	GGCATGGACTGTGGTCATGAG	TGCACCACCAACTGCTTAGC
**CCL2**	GTCTCTGCCGCCCTTCTGT	TTGCATCTGGCTGAGCGAG
**MYC**	GTGCAGCCGTATTTCTACT	TCTTCCAGATATCCTCGCT
**MINA**	GGTGTGTCAATGGGAAGA	TCTCTGAGGTTGGTGAAAC
**NCOA1**	AGAATAAACCGCCAGCAGAG	CAATCTGACTTCCGGGTGAG
**ESRRA**	CAGGCCACAAGGAAGAGGAG	CTCACAGGATGCCACACCAT
**KLF10**	TTCTGTGCCTTCCTCAGAGA	TGGAGAGACCAACACTGACT
**ATR**	CATGTTTGAAGACGGTGTGC	CATACCCAGCTGGCACAAAT
**CYB5R4**	GTCAGCCCTTATATGGAGTA	AGTTCAGTACCATCTGATCC
**ENDOG**	CCAGAATGCCTGGAACAA	CTGTGCAGACATAGACGTT
**NMI**	GACAGGAATGGAAGGCAT	CATTCTTTGCCCGTTGAAA
**APEX1**	TCTCGCGAGTAGGGCAACGC	TCTTCCGCCACCGCTCCCTT
**TNF**	GCCCAGGCAGTCAGATCATC	GGGTTTGCTACAACATGGGCT
**IL8**	GTTTTTGAAGAGGGCTGAGAATTC	CATGAAGTGTTGAAGTAGATTTGCTTG
**47S**	CGCCGCGCTCTACCTTACCTA	TAGGAGAGGAGCGAGCGACCA
**28S**	TGTCGGCTCTTCCTATCATTGT	ACCCAGCTCACGTTCCCTATTA
**ELK1**	AGAACATCATCCGCAAGGTGA	GGTAACAGACACCTCTGGCT
**GABPA**	AAGAACGCCTTGGGATACCCT	GTGAGGTCTATATCGGTCATGCT
**NIP7**	CCGGGTGTACTATGTGAGTGAGAA	TTGTGGGTTTTAGTGAATTTTCCA
**RPS15A**	AATGTCCTGGCAGATGCTCTCAAG	GAGCACGGCCTAATAAGCACCTG
**RPL35**	GCAGGAACAGAAAGACCA	CCCAAAACCTACCAGTGTCA
**RPL27**	TCGCCAAGAGATCAAAGATAA	CTGAAGACATCCTTATTGACG
**RPS19**	GTGAACCAGCAGGAGTTCGT	GGAGGTACAGGTGCCGTG
**NRF1**	GAGGATGATCCTGGAAGACC	AATTCCGTCGATGGTGAGAG
**TFAM**	TGCTAAAGAGGACGAAACTCG	ACGTAGAAGATCCTTTCGTCCA
**TFB2M**	AACCACCTGCTATGTCTTCTCG	TGTCTGCTGTCCAAGGAACT
**NDUFB9**	CTACCTGACCCATCAGCAAAA	AGCAAAGTATCGGTATTTGTCTCTC
**NDUFB5**	AGCTGGAAGTGCGAAAATTG	GGAGTTGCTTTCGGAGAATG

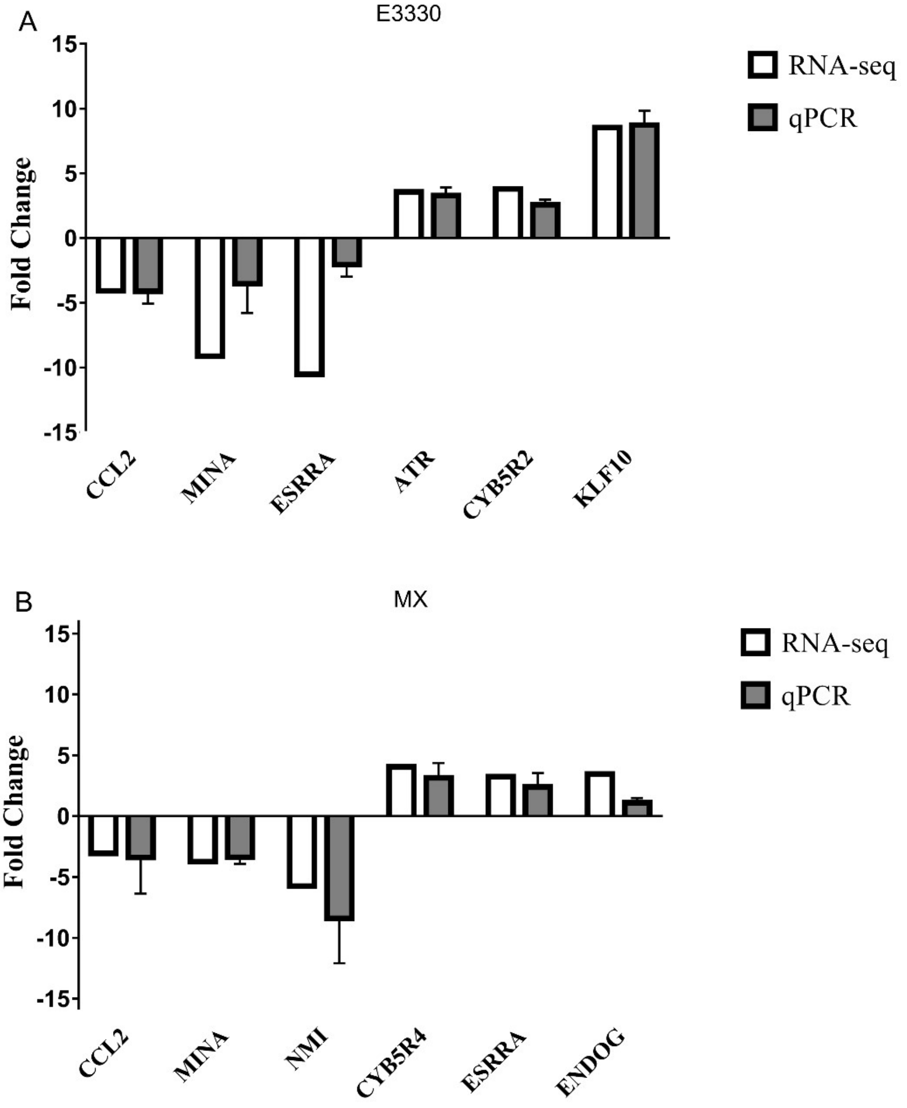

Fig S1. Expression analysis using the techniques of qPCR compared to RNA-seq. Eight differentially expressed genes belonging to various functional categories were selected for qPCR validation after (A) MX and (B) E3330 treatment during inflammation.

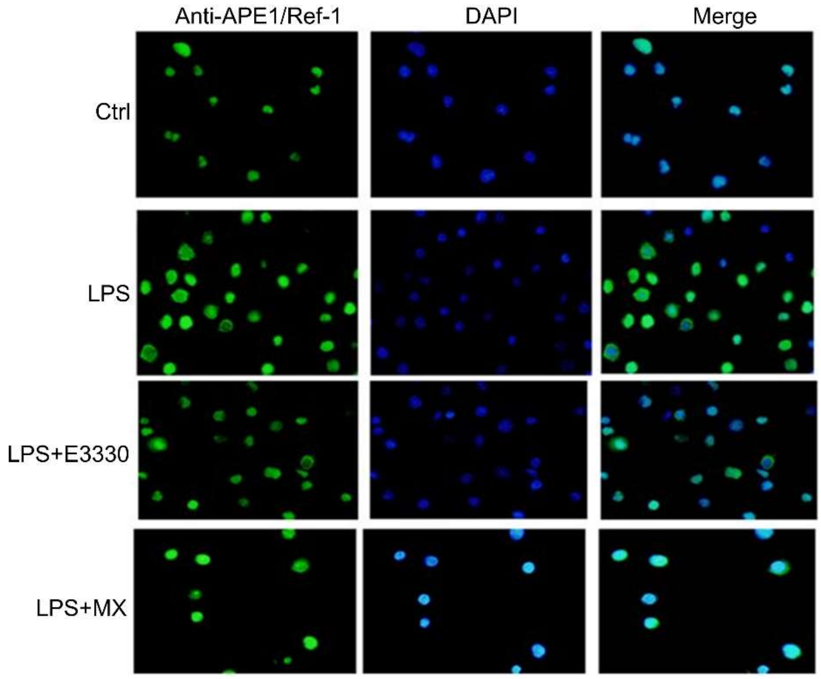

Fig S2. Immunofluorescence of APE1-Ref-1 cellular localization in U937 cells. Green fluorescence – APE1; Blue fluorescence – DNA stain; Merge- superposition of images. The LPS treatment induces the translocation of APE1 from the nucleus to cytoplasm and the LPS/MX treatment reduces the protein fluorescence at the cytoplasm.

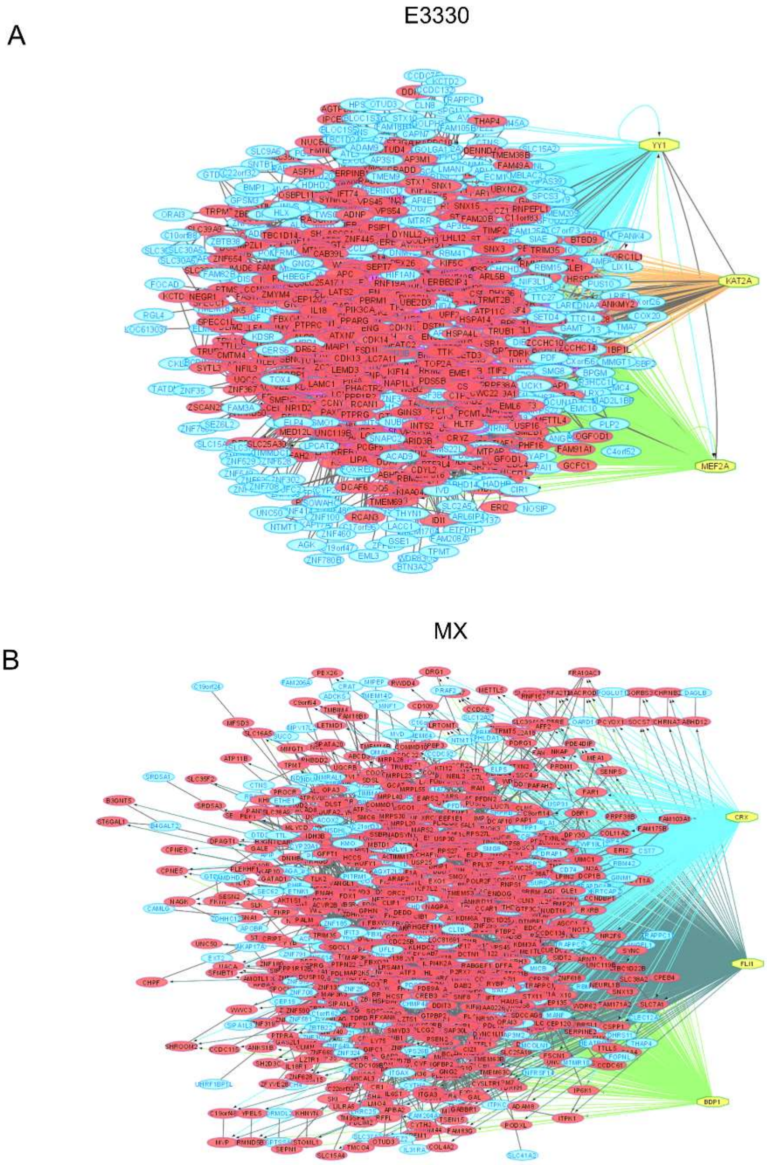

Fig S3. Protein-protein interaction (PPI) network formed by the up-regulated genes. Mater regulators predicted by iRegulon are highlighted in yellow, targets of the master regulators are shown in red. In blue, genes are not targets of the master regulators. (A) PPI network determined from up-regulated gene list upon E3330 treatment using STRING software and analyzed by Cytoscape. (B) PPI network of up-regulated genes after MX treatment

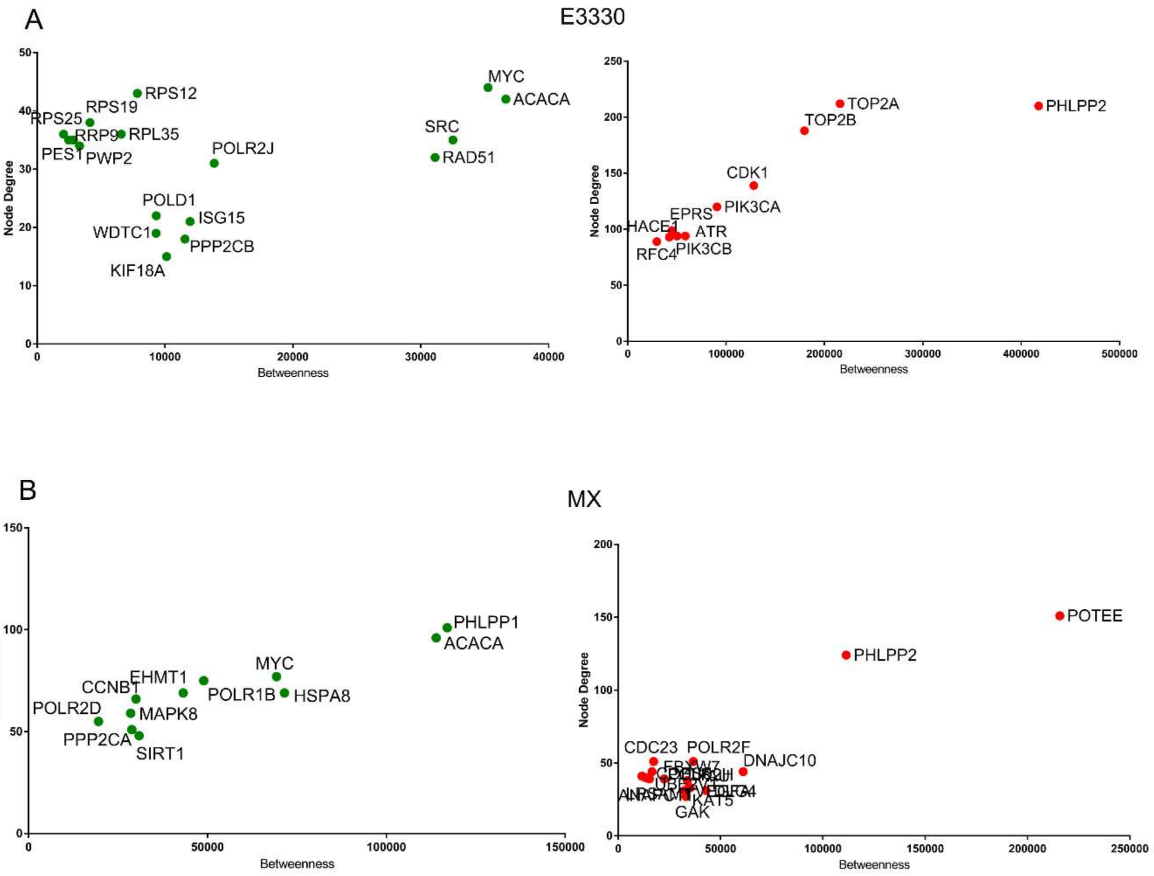

Fig S4. Hub-bottleneck genes in networks formed by differentially expressed genes. (A) Top 10 genes with higher node degree value and top 10 with higher betweenness value, calculated by CenTiScape plugin of up (left) and down (right) E3330 networks. (B) Top 10 genes with higher node degree value and top 10 with higher betweenness value, calculated by CenTiScape plugin of up (left) and down (right) MX networks.

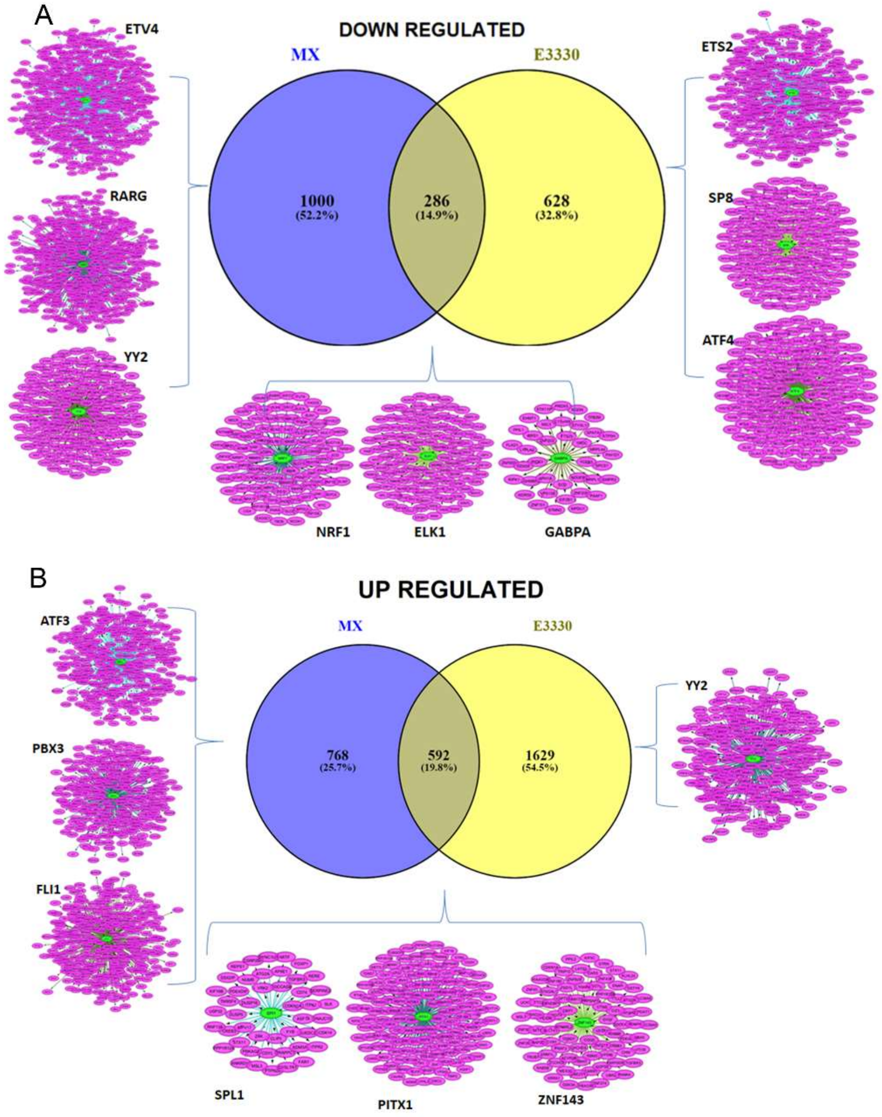

Fig S5. Main regulators of commons and exclusive genes differentially regulated by E3330 and MX. (A) Venn diagram represented commons and exclusive down-regulated genes and his main regulators. (B) Venn diagram represented commons and exclusive up-regulated genes and his main regulators.
